# The mechanisms and factors that induce trained immunity in arthropods and mollusks

**DOI:** 10.3389/fimmu.2023.1241934

**Published:** 2023-09-07

**Authors:** Mingming Zhao, Zhongyang Lin, Zhihong Zheng, Defu Yao, Shen Yang, Yongzhen Zhao, Xiuli Chen, Jude Juventus Aweya, Yueling Zhang

**Affiliations:** ^1^ Institute of Marine Sciences and Guangdong Provincial Key Laboratory of Marine Biotechnology, Shantou University, Shantou, China; ^2^ College of Ocean Food and Biological Engineering, Fujian Provincial Key Laboratory of Food Microbiology and Enzyme Engineering, Jimei University, Xiamen, Fujian, China; ^3^ Guangxi Academy of Fishery Sciences, Guangxi Key Laboratory of Aquatic Genetic Breeding and Healthy Aquaculture, Nanning, China

**Keywords:** trained immunity, arthropods, mollusks, metabolism, epigenetic modifications

## Abstract

Besides dividing the organism’s immune system into adaptive and innate immunity, it has long been thought that only adaptive immunity can establish immune memory. However, many studies have shown that innate immunity can also build immunological memory through epigenetic reprogramming and modifications to resist pathogens’ reinfection, known as trained immunity. This paper reviews the role of mitochondrial metabolism and epigenetic modifications and describes the molecular foundation in the trained immunity of arthropods and mollusks. Mitochondrial metabolism and epigenetic modifications complement each other and play a key role in trained immunity.

## 1 Introduction

The immune system evolved under coevolutionary selection and is the backbone of animal resistance to pathogen attack (1). The immunity of an organism is divided into adaptive immunity and innate immunity. Adaptive immunity evolved independently in vertebrates (2) and is the only one that has memory. However, a growing number of studies have shown that innate immunity can enhance immune responses to secondary infection, which imply that innate immunity has memory (3). However, unlike adaptive immune memory, the memory of innate immunity involves epigenetic modification (4).

**Figure 1:**
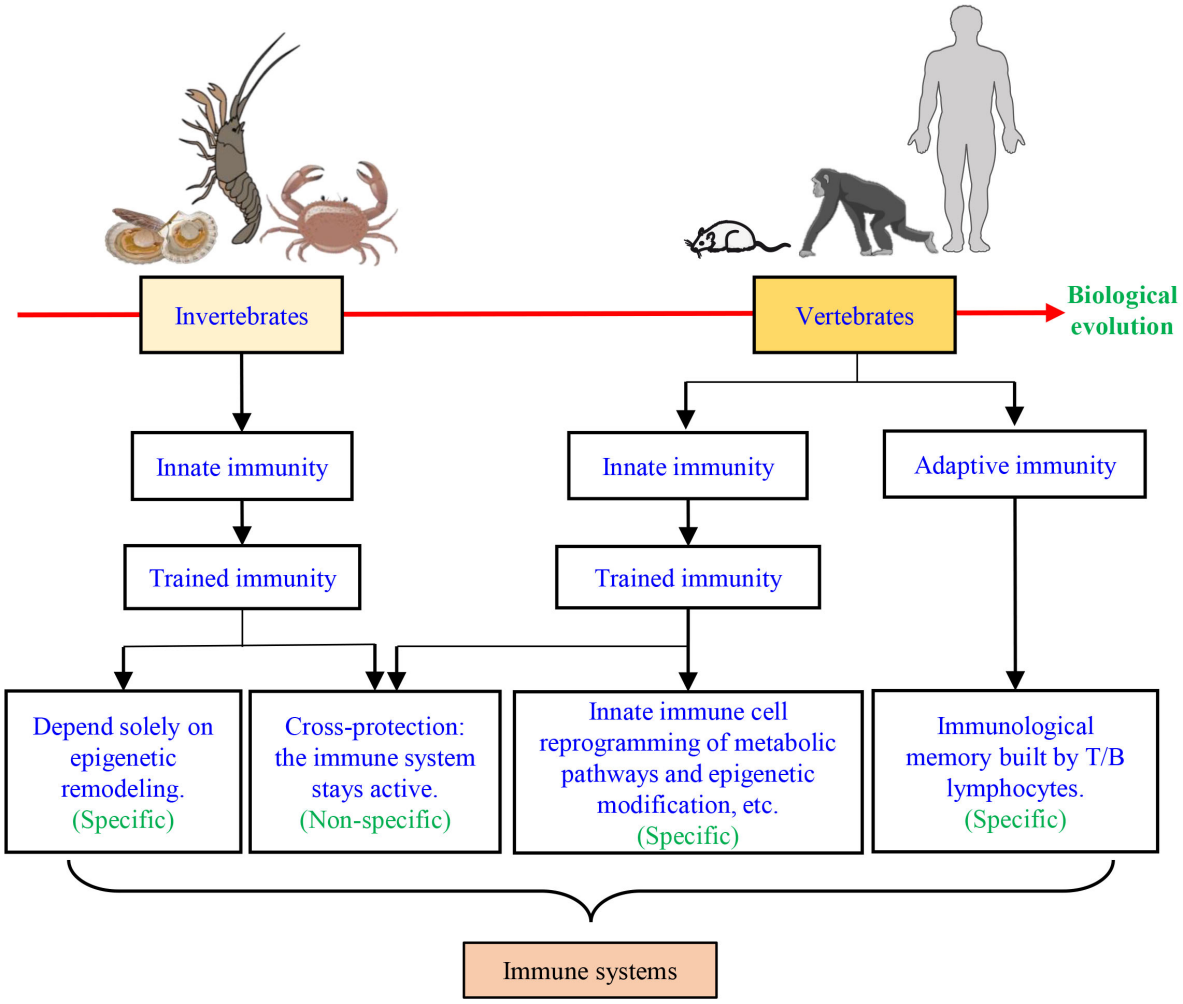
The immune systems in vertebrates and invertebrates.

In vertebrates, besides adaptive immune memory, innate immune memory or trained immunity has been described (5, 6). The ability of the vertebrate innate immunity to build immunological memory in macrophages was first described in 1986 (7), which seems to result from environmental stress conditions (8–10), and therefore is distinct from classical immunologic memory triggered by the T or B lymphocytes (11, 12) (**Figure 1**). Many studies on vaccines and pathogens have provided evidence of innate immune memory, such as in SCID mice, which have no T/B lymphocytes, have revealed that Bacille Calmette-Guerin (BCG) could still protect against disseminated candidiasis (13), indicating that some vaccinations and even infections can induce more broad protection against other pathogens through trained immunity mechanisms (5, 12). In vertebrates, trained immunity may improve protection against emerging pathogens and also against future new pandemics (14).

In the last couple of years, trained immunity in mammals has been extensively reviewed (6). Infection and/or vaccine increases the efficiency of immune responses or enhances the resistance to reinfections by related and unrelated pathogens, i.e., offers a cross-protection (15–17). Since the adaptive immune system of vertebrates remembers previous encounters and mounts a robust immune response (18), vertebrates are not ideal models for research into innate immune memory. Recent studies have shown that organisms, such as invertebrates and plants, which lack adaptive immunity, show greater protection from reinfection (19–21). For instance, *Anopheles albimanus*, *Marsupenaeus japonicus, Crassostrea gigas*, etc., are reported to build immunological memory to reinfection by the same and different pathogens (22–24).

Among metazoan species that number around 1,162,000, about 1,112,000 (about 95.70%) are invertebrates (25). Given that invertebrates lack lymphocytes and are thought incapable of developing immune memory (26), coupled with the species richness of invertebrates, they are the ideal models for studying innate immune memory. Moreover, many invertebrate species provide steady sources of food globally (27), such as shrimps, scallops, crabs, abalone, etc., are farmed on a largescale (28–31). Thus, understanding how these organisms enhance their immunity, such as via trained immunity, would go a long way toward improving their aquaculture. Therefore, this current review brings together information on trained immunity in invertebrates, especially those that serve as food sources, to understand better how these organisms protect themselves from repeated infections. The concepts and mechanisms from emerging scientific fields will open new avenues for cultivating new species and enhancing disease prevention and treatment of aquaculture animals.

## 2 Trained immunity in arthropods and mollusks

**Figure 2:**
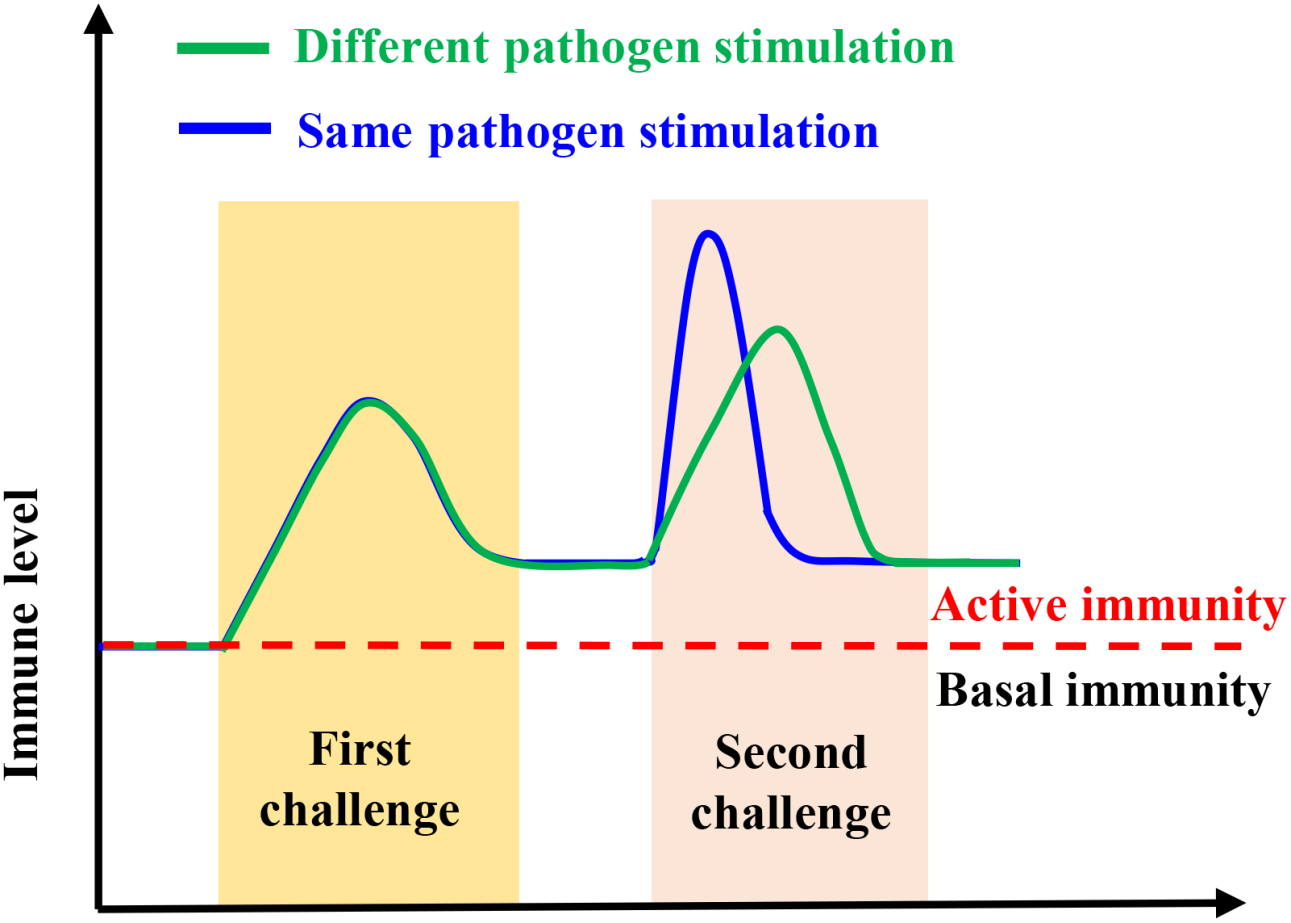
The model of trained immunity.

The innate immunity of invertebrates displays some features of an immunological memory (32), which has the same function as the vertebrate adaptive immune system (15, 16). In arthropods and mollusks, trained immunity has been reported in many, including Brine Shrimp (*Artemia*), the Peruvian scallop (*Argopecten purpuratus*), Chinese mitten crab (*Eriocheir sinensis*), Pacific oyster (*Crassostrea gigas*), kuruma shrimp (*Marsupenaeus japonicus*)*, etc.* (22, 23, 29, 30, 32). Many studies have shown that the innate immunity in organisms that have or lack adaptive immunity can mount increased resistance to reinfection through innate immunity memory or trained immunity (33–36). For instance, innate immune memory is induced by microbiota to protect mosquitoes against *Plasmodium* (37), while *Bombus terrestris* can protect themselves against different pathogens through innate immune memory (38). Similarly, innate immune memory has been identified as an immune defense mechanism in snails (39). In addition to forming memories to the same pathogen, trained immunity helps the host to resist infection by other pathogens, i.e., providing a cross-protection (40) (**Figure 2**). This phenomenon is because the immune system stays active after the first stimulus, which does not recover to the base level before the next infection (6), indicating that trained immunity also has some specificity (**Figure 1**). In invertebrates, pattern recognition receptors and/or the genetic diversity of immune molecules and the functional diversity of immune proteins are believed to provide the basis for trained immunity (36, 41–43).

Growing evidence indicates that the molecular mechanisms of trained immunity are epigenetically regulated (17, 44, 45) but not through mechanisms dependent on T and B cell adaptive responses (46). Some mechanisms shown to modulate trained immunity, including histone acetylation, DNA methylation, modulation of microRNA, and noncoding RNA expression (5, 47–49), are defined as epigenetic modifications (50). Innate immune cell reprogramming of metabolic pathways is another basis for forming a trained immunity (51). When innate immune cells are exposed to the first pathogen stimulus, metabolic pathways and epigenetic modifications occur, providing rapid and enhanced immune response upon subsequent pathogen challenge (5, 52). Thus, the trained immunity of arthropods and mollusks could also be affected by epigenetic modifications and metabolic reprogramming, which provides a broader pattern of specificity and immune memory.

## 3 The basis of trained immunity in arthropods and mollusks

During an immune response, host cells’ surface receptors recognize pathogens and transmit the infection signal to the cellular signal transduction pathway to activate target genes’ expression, followed by the release of effector proteins or factors to clear the pathogen. Thus, pathogen recognition initiates the immune response, hence, differences in this recognition induce different immune responses.

Trained immunity has immune memory and primitive specificity to the same pathogen, probably due to receptor diversity (53). The innate immune system’s pattern recognition receptors (PRRs) recognize different microbial species and mediate a broad specificity pattern in vertebrates (53, 54). Similarly, invertebrates have evolved genetic mechanisms capable of producing thousands of different immune proteins from a few genes, which helps them to clear a wide range of pathogens (55). For instance, *Tribolium castaneum*, *Anopheles gambiae, Drosophila melanogaster*, and *Bombus terrestris*, etc. diversify their immune genes’ sequence to enable them to exert a certain degree of specificity to microbial pathogens (38, 56–58).

The ability of invertebrates to discriminate between pathogens is based on a set of PRRs specific for pathogen-associated molecular patterns (PAMPs) of different pathogens (59). Many immune gene families of these PRRs in invertebrates could mediate the non-specific immune response (17, 60, 61), which could also be a form of trained immunity whereby gene expression to synthesize immune proteins is induced by environmental changes. For example, the Down syndrome cell adhesion molecule (Dscam), which is well studied in invertebrates, has been shown to play a role in mounting adaptive-like immunity by specific splicing to produce different immune protein isoforms during pathogens stimulation (62–66). Similarly, C-type lectin-like domain (CTLD) proteins, which perform important tasks in immunity by acting as PRRs (67) and as effector proteins with bactericidal activity (68, 69), are expressed in the genomes of many organisms, including, cephalochordata, echinodermata, insecta, nematoda, cnidaria, porifera, and placozoa (70–76). In penaeid shrimp, such as *Litopenaeus vannamei*, the C-terminal domain of hemocyanin contains a highly variable genetic sequence that is structurally homologous to immunoglobulin (Ig) and can recognize and bind with bacteria or red blood cells to agglutinate or cause hemolysis (77). The C-terminus of hemocyanin also possesses single nucleotide polymorphisms (SNPs), which is related to shrimp’s resistance to different pathogens (78, 79). In addition, many multigene family proteins in invertebrates, such as npr-1, Sp 185/333 protein, NLRs, TLRs, Caspase gene, and fibrinogen-related proteins (FREPs), are immune effectors and/or modulators of cellular processes involved trained immunity (17, 37, 80–87) (**Table 1**).

**Table 1:** Diversity of immune-related genes and proteins in invertebrates.

Organism	Species	Protein/gene	Family/number of genes	Tissue	Stimuli	Function	References
Sponges	*Amphimedon queenslandica*	NLRs	Family of pattern recognition receptors			PRRs	(86)
Echinoderms	*Strongylocentrotus purpuratus*	Sp185/333	16 genes and 80 full-length transcripts	Coelomic fluid, axial organ, gut, esophagus, gonad, and pharynx	LPS	Syncytium formation	(88)
	*Strongylocentrotus purpuratus*	Sp 185/333	Sp 185/333 gene family		LPS or *Vibrio diazotrophicus*	Antimicrobial activity	(85)
	*Strongylocentrotus purpuratus*	Sp 185/333	Sp185/333 protein				(82)
	*Strongylocentrotus purpuratus*	TLRs	222 TLR genes	Sperm		Mediates the alternative and lectin complement pathways	(87)
Arthropods	*Daphnia magna*	Dscam	13,000 different transcripts	Whole-body		PRRs	(89)
	*Anopheles gambiae*	Dscam	Pathogen-specific splice variants		LPS, PGN	PRRs	(90)
	*Anopheles gambiae*	Dscam	Pathogen-specific splice-forms	Whole-body	*Plasmodium falciparum*	PRRs	(65)
	*Litopenaeus vannamei*	Hemocyanin	Hemocyanin fragments	Hemolymph	*Vibrio alginolyticus*, *V. fluvialis*	Bacteria agglutination	(91)
	*Litopenaeus vannamei*	Hemocyanin	Hemocyanin SNPs	Hemolymph	*V. parahaemolyticus*, *V. alginolyticus*, *V. harveyi*, *V. fluvialis*, *V. anguillarum*, *Aeromonas hydrophila*, *A. sobria*, *Pseudomonas fluorescens*, *Staphylococcus aureus*	Antimicrobial protein	(77)
	*Scylla serrata*	Hemocyanin	Hemocyanin subunits (70, 72, 75, 76, 80 kDa)	Hemolymph	*V. parahaemolyticus*, *V. alginolyticus*, *V. harveyi*, *V. fluvialis, A. hydrophila*, *S. aureus*	Agglutination activities	(92)
	*Litopenaeus vannamei*	Hemocyanin	12 O-glycosylationsites	Hemolymph	*V. parahaemolyticus*, *S. aureus*	Agglutination and antibacterial activities	(93)
	*Litopenaeus vannamei*	C-terminus of Hemocyanin	13 SNPs	Hemolymph	*V. parahaemolyticus*	Agglutinative activities	(78)
	*Litopenaeus vannamei*	Hemocyanin	3 variant sequences of the hemocyanin subunit	Hepatopancreas	*Escherichia coli* K12, *V. parahaemolyticus*, *V. alginolyticus*, *V. fluvialis*, *Streptococcus pyogenes, S. aureus*	Agglutination activities	(94)
	*Marsupenaeus japonicus*	Caspase	203 caspase genes	Hemolymph hepatopancreas, muscle, gill, and intestine	*V. parahaemolyticus*, WSSV	Enhances virus-induced apoptosis	(95)
Molluscs	*Crassostrea virginica*	CTLD	CTLD gene families		*Alliroseovarius crassostreae*	PRRs	(61)
	*Crassostrea gigas*	TLRs	83 TLR genes	gill	*V. anguillarum*, *V. tubiashii*, *V. aestuarianus*, *V. alginolyticus*	PRRs	(60)
	*Biomphalaria glabrata*	FREPs	Putative immune repertoire.	Haemolymph		Phagocytosis or encapsulation	(80)
Nematodas	*Caenorhabditis elegans*	CTLD	283 gene family members.			PRRs	(53)
	*Caenorhabditis elegans*	npr-1	npr-1 mutant.	Whole-body		p38 MAPK signaling	(84)

NLRs, NOD-like receptors; PRR, pattern recognition receptor; LPS, lipopolysaccharide; PGN, peptidoglycan; TLRs, Toll-like receptors; Dscam, Down syndrome cell adhesion molecule; SNPs, single nucleotide polymorphisms; CTLD, C-type lectin-like domain; FREPs, fibrinogen-related proteins; Npr-1, neuropeptide Y receptor gene.

Gene evolution could result from the interaction between hosts and pathogens, indicating that changes in gene expression patterns could be a response to external environmental pressure resulting from long-term evolution. Thus, specific immune pathways and common immune pathways are preserved in the course of evolution. The immune specificity of invertebrates is mainly based on the diversification of somatic gene sequences that encode recognition molecules, effector-enhancement molecules, and other immune molecules (96–98), synonymous with receptor diversification in the adaptive immune system of vertebrates (95). In Arthropods, gene sequence diversity prevents different pathogens from interfering with their immune response (**Table 1**). The evolutionarily conserved immune system components might explain simple forms of specific immune memory from trained immunity, such as the degree of specific immune reactions of the Toll and Imd pathways (99).

In some invertebrates, macromolecular proteins undergo specific degradation and modifications to allow them to respond to different pathogens. For instance, in response to pathogenic bacteria, the hemocyanin protein of penaeid shrimp degrades into functional peptides to enhance their antimicrobial immunity (100–103) (**Table 1**). Therefore, arthropods and mollusks do not only encode immune genes but also immune effector molecules with different molecular polymorphisms, which constitute part of their trained immunity (**Table 1**). Similarly, other invertebrates have diversified their immune gene sequences and repertoire of diversified receptors (**Table 1**).

## 4 The effect of metabolic and epigenetic modification on trained immunity in arthropods and mollusks

The immune system of organism senses and responds to environmental stress, which is high-energy demanding. Trained immunity is associated with many metabolic pathways to increase the ability of immune cells to respond to secondary infections through metabolic reprogramming (104). Environmental cues can also change chromatin structure through epigenetic modification, which could be passed on to the next generation to facilitate adaptation (105). Current studies show that epigenetic modifications, including histone modifications, DNA methylation, chromatin remodeling, and non-coding RNA (106, 107), operate to maintain cell identity (108–110). The mechanism of epigenetic modifications can play an important role in host-pathogen interactions by regulating gene expression (111–116), indicating that metabolic and epigenetic modification are two essential parts of trained immunity.

### 4.1 Cellular metabolism

The cellular immunity of invertebrates mediated by hemocytes consists of inflammatory responses, includes phagocytosis, encapsulation, cytotoxicity, and synthesis or release of microbicidal agents (117). Different immune signals induce different cellular metabolic reorganizations, which are critical for the epigenetic modifications in trained immunity (33, 52, 118). Given that immune responses are high-energy processes, metabolism provides energy to maintain cellular hemostasis and enhances immune cells’ functions (51). For instance, the metabolic product lactate can inhibit the activity of histone deacetylase (HDAC) to increase gene accessibility (119). Moreover, mitochondria are key factors that control many epigenetic enzymes (120). For instance, the activities of alkaline phosphatase, alanine aminotransferase (ALT), phenoloxidase, acid phosphatase (ACP), and lactate dehydrogenase (LDH), peaked at 6-12 h after injection with fungal spores of *Spodoptera littoralis*, while the highest immune responses and intermediary metabolism occurred 12 h post-injection (121). Similarly, ACP and ALP are the hydrolytic enzymes that mediate the dephosphorylation of nucleotides, proteins, and alkaloids and have been implicated in lipid hydrolysis to provide energy for resistance to external stimuli (122). Thus, metabolic change can promote epigenetic reprogramming under inflammatory stimulation to achieve trained immunity phenotype (5, 33).

During immune stimulation, the metabolic state of cells is modulated to regulate the expression of different genes by retrograde signaling in the mitochondria to promote different cellular functions, such as differentiation, adaptation to challenge, etc. (123, 124). However, under an inappropriate metabolic state, such as due to an effect in the electron transport chain, remedial measures are taken to maintain the production of certain tricarboxylic acid cycle (TCA) intermediates by glutamine-dependent reductive carboxylation (125). The mutual regulation between metabolism and genes expression have been observed in some invertebrates, such as Ca*enorhabditis* elegans, *Daphnia pulex*, *L. vannamei, Argopecten purpuratus*, *Scylla paramamosain*, etc. (29, 126–129).

**Figure 3:**
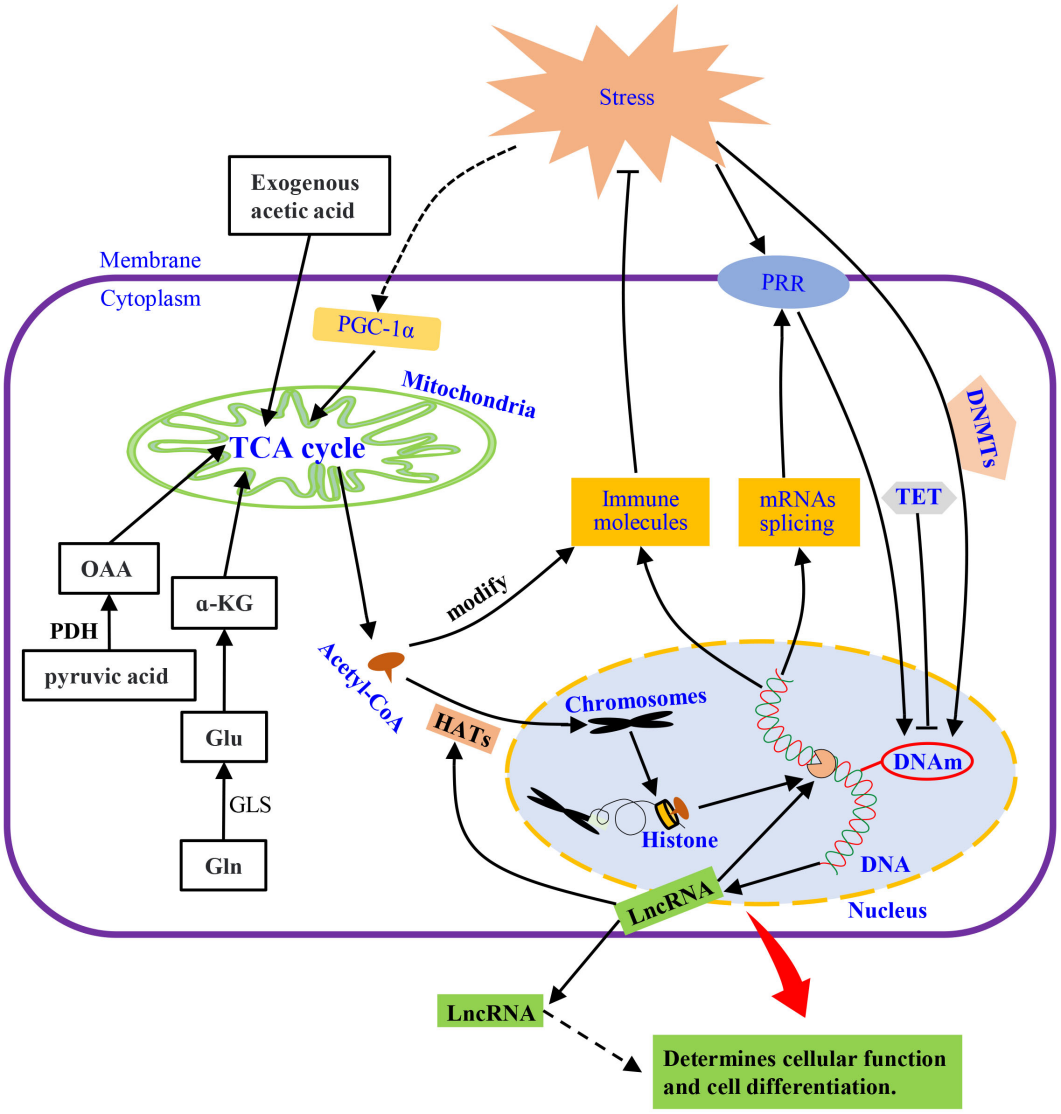
The mechanisms of innate immunity. Under external stimulation, metabolism and membrane receptors directly or indirectly affect DNA methylation, which induces the synthesis of immune molecules by regulating transcription and translation or releases spliced mRNAs. LncRNA is also released to modulate transcription and intercellular information transmission. The external stimuli also activate PGC-a, regulate the TCA cycle, and acetylate chromatin histones through its intermediate product, acetyl-CoA, to promote transcription. Pyruvate, glutamine, or exogenous acetic acid metabolism, supplement the substances needed for the TCA cycle to keep it running. HATs, histone acetyltransferases; α-KG, α-ketoglutarate; PGC-1α, peroxisome proliferator-activated receptor γ coactivator 1α; DNMTs, DNA methyltransferases; TET, Ten-eleven translocation; DNAm, DNA methylation; LncRNAs, Long Non-coding RNAs; OAA, oxaloacetic acid; PDH, pyruvate dehydrogenase; GLS, glutaminase; PRR, pattern recognition receptor; Gln, glutamine; Glu, Glutamate.

Mitochondria provide metabolic intermediates and their derived products, e.g., S-adenosyl methionine (SAM) and acetyl-CoA, which drives epigenetic modification, such as histone acetylation by acetyl-CoA (120, 130). Levels of acetyl-CoA affects the activity of histone acetyltransferases (HATs) to regulate gene expression by changing the acetylation of the whole histone (131, 132), which is highly dependent on fatty acid metabolism and glucose availability in mitochondria (131, 133, 134) (**Figure 3**). Thus, histones acetylation drives the epigenetic control of gene expression through transcriptional programs (135, 136). For instance, exogenous acetate can produce acetyl-CoA to maintain global histone acetylation when acetyl-CoA production by ATP citrate lyase (ACLY) is limited (137). This complementary mechanism relies on two important anaplerotic mechanisms, i.e., the conversion of pyruvate to mitochondrial oxaloacetate by pyruvate decarboxylase and the conversion of glutamate by activation of glutaminolysis and subsequently to α-ketoglutarate (α-KG) (123). However, mitochondria dysfunction induced by exposure to environmental mutagens or pathogen stimulation can suppress mitochondrial oxidative metabolism in invertebrate (138, 139), which could be responsible for the change in gene expression profiles after pathogen challenge (140–142).

An increase in energy metabolism can be regulated by peroxisome proliferator-activated receptor γ coactivator 1α (PGC-1α) to alter cellular energy demand in different tissues upon activation by stimuli (143, 144). In addition, PGC-1α can coordinate tissue-specific transcription to mediate the plasticity of cells (145) (see **Figure 3**). These cellular events could be modulated by PGC-1α through steroid receptor coactivator 1 (SRC-1) and CREB-binding protein (CBP) to affect the HAT complex (146). Thus, the influence of immune response through metabolism could affect some aspects of inflammatory or autoimmune diseases.

### 4.2 Epigenetic modification

#### 4.2.1 Histone acetylation

Nucleosomes are formed when DNA strands are wrapped around eight core histones and then compressed into chromosomes (147). Intrinsically, the compact structure of nucleosomes is repressive regarding unwanted transcription activity. Histone proteins can be modified post-translationally at various residues through methylation, acetylation, phosphorylation, and/or ubiquitination, which changes the chromatin structure (148–150). The increased resistance of *Artemia* to *Vibrio campbellii* infection, mediated by increased acetylation levels of histone H3 and H4, could be passed on to the next generation (151). Histone acetylation is a reversible process involving the addition/removal of an acetyl group on lysine residues catalyzed by HAT/histone deacetylase (HDAC) (152, 153) (**Figure 3**). Although histone deacetylation catalyzed by HDAC tightens the chromatin structure to silence gene expression (153), pathogens infection can disrupt this structure to inhibit host gene expression. For instance, the ICP11 protein of white spot syndrome virus (WSSV) can damage the nucleosome assembly in *Penaeus vannamei*, by binding to histone proteins to evade the hosts’ immune responses to promote viral replication (154).

#### 4.2.2 DNA methylation

Various gene expression changes are induced in the immune system during pathogen infection, which involves regulating epigenetic mechanisms. For instance, DNA methylation, a kind of epigenetic modification, plays an important role in various biological processes (155). DNA methylation is a stable covalent cytosine modification in a cytosine-guanine dinucleotide (CpG) context, which in the genomes of vertebrates, are highly methylated but sparsely methylated in invertebrates (156). This genomic methylation is regulated by DNA methyltransferases (DNMTs), a key mechanism for controlling gene expression in various organisms (157, 158) (**Figure 3**). The oxidation of 5-methylcytosine (5mC) at CpG dinucleotides to form 5-hydroxymethylcytosine (5-hmC) serves as an epigenetic marker (159) because 5-hmC is enriched at the enhancers of most highly transcribed genes (160). Conversely, the promoters of highly expressed genes that are highly expressed are loss of CpG methylation and form CpG islands (161). Therefore, CpG islands are important for chromosome stability (162, 163).

In *Drosophila melanogaster* and *Aedes aegypti*, genomic hypermethylation caused by Wolbachia infection may be associated with the up-regulation of DNA methyltransferases (DNMTs) gene expression (116, 164, 165). Under these conditions, the cytosine-5 of the host insect DNMTs are induced under bacterial infection to affect the expression of antimicrobial peptides (AMPs) (166). In *Drosophila*, DNMTs are also required for antiviral innate immune responses (167). Since DNA hypermethylation has been linked to transcriptional silencing (163), it is a key and stable mechanism for repressing gene transcription (168), whereas hypomethylation may increase the transcript levels of genes (169). DNA methylation is a reversible process that can be reversed by Ten-eleven translocation enzymes (TET) when gene expression is active (170). Therefore, the interplay between TET proteins and DNMTs controls the DNA methylation landscape (**Figure 3**).

The methylation of most DNAs in invertebrates is not in the intergenic regions (171, 172) but rather in genomic loci that match small RNAs in gene bodies, which are densely methylated, probably because they regulate the transcription and mRNA splicing of target genes (173). For instance, the DNA methylation in *Apis mellifera* controls the alternative splicing of mRNA and is involved in gene expression (174, 175). Pathogens may establish successful infection by manipulating the expression of host genes via DNA methylation. In *Bombyx mori*, cytoplasmic polyhedrosis virus infection may lead to hypermethylation of the p53-2 gene, suppressing its expression to facilitate the proliferation of infected cells (169).

Although histone and DNA methylation are reported widely in most invertebrates, such as arthropods and mollusks, how histone and DNA methylation interact to regulate gene expression and induce trained immunity in invertebrates remains unknown.

#### 4.2.3 Non-coding RNA

Long non-coding RNAs (LncRNAs) are involved in various regulatory functions in animals, including gene regulation at multiple levels, such as at the post-transcriptional levels, enhancers, promoters, and chromatin modification complexes (176, 177). For example, lncRNAs can affect promoter activity or mRNA translation (178) and form miRNA precursors to regulate target gene expression (152). LncRNAs do not only act intracellularly by regulating HDAC, CBP/P300, and HAT but also can be transported to other cells by exosomes (179, 180), which could be one of the mechanisms of epigenetic inheritance across generations (**Figure 3**). Both lncRNAs (*A. aegypti*) and microRNAs (*C. elegans*) show genetic characteristics (181, 182). Thus, in invertebrates, various studies have reported on the regulation of transcriptional activity by non-coding RNAs, such as miRNAs in *Galleria mellonella*, let-7 miRNA cluster in silkworms (*Dazao P50*), miRNAs/lncRNAs in *A. aegypti*, microRNA-8 in *Drosophila*, miRNA-317 in *C. gigas*, etc. (183–188).

## 5 Heritability of trained immunity

Adaptive immune memory can last for a long time and respond quickly to reinfection by the same pathogen. Invertebrates such as *Artemia* display trained immunity with similar features as adaptive immunity (32). The memory of innate immunity could persist for days or almost the entire lifetime, and in some cases across generations (181, 182, 189–191). Trained immune memory induced by inactivated bacterial and viral antigens has been reported in shrimp and crayfish, the main manifestation of which is that the secondary immune response is greatly improved compared with the control (192). For instance, the offspring of *Trichoplusia ni* from parents raised on a bacteria-rich diet had an increased expression of immune-related genes and immune enzyme activity (193). Similarly, the initiation of transgenerational immunity occurs after the red flour beetle *Tribolium castaneum* is exposed to heat-killed bacteria (194). Early life microbial exposure improves oyster survival when challenged with the pathogen causing Pacific oyster mortality syndrome (POMS) in both the exposed generation and subsequent generation (195). When challenged with specific bacteria, *Artemia* could acquire strain-specific immunity that increases resistance against the same strain of bacteria and is transmissible to the progenies of successive generations (196, 197).

From the foregoing, it is clear that trained immunity can also respond specifically to external stimuli and be passed on to the next generation. The transgenerational effects of trained immunity induction in vertebrates have been confirmed (198–200). Moreover, the mechanism of trained immune memory in vertebrates could be mediated by innate immune cells, such as monocytes, macrophages, and natural killer cells (201). Given that invertebrate immunocytes perform the same immune functions as vertebrate macrophages (202), a similar memory mechanism might also be present in invertebrate immunocytes, although the life span of immune-functioning cells in blood is shorter than that of trained immunity. Therefore, more research is needed to further unravel the mechanisms of trans-generational immune priming (TGIP) in invertebrates during trained immunity memory.

## 6 Application prospects of trained immunity

Invertebrates lack an adaptive immune system and are an excellent model for studying innate immune defense mechanisms (203). For instance, Drosophila has been used as a valuable insect model to study immune mechanisms of neurodegenerative diseases, such as *Alzheimer’s* disease and *Parkinson’s* disease (204, 205).

In aquaculture animals, trained innate immunity has been reported in mollusks, such as oysters and abalone (23, 31, 206, 207). For instance, DNA methylation patterns are found to vary with changes in seasons (especially in temperature) in the oyster *Isognomon alatus* (208, 209) or changes in ocean acidification and salinity in the *Haliotis discus hannai* (210, 211). Similarly, the speed of water currents can change DNA methylation patterns, as in the snail *Potamopyrgus antipodarum* (212, 213). Generally, hypomethylation occurs in different oyster species after infection with toxic algae (214, 215). In the freshwater gastropod *Biomphalaria glabrata*, Trematode infection induces DNA methylation machinery proteins to impact DNA methylation levels (216, 217). Exposure to air has also been reported to affect innate immunity and DNA methylation in *M. japonicus* (218). Thus, environmental and biological factors can influence DNA methylation levels in arthropods and mollusks, laying a foundation for the selective breeding of economic species of invertebrates. Although elucidating the epigenetic mechanisms in these species is of great significance to genetic breeding, there is still limited knowledge on pathogen-host interactions, which is one factor limiting trained immunity application for economically important arthropodan and molluscan species.

Prophenoloxidase (ProPO) and transglutaminase (TGase) genes, which play crucial roles in melanization and coagulation, are important constituents of the innate immune system of arthropods that protect the host from invading pathogens (219, 220). For instance, in penaeid shrimp, hemocyanin protein interacts with TGase to modulate its expression, affecting hemolymph clotting (221). Similarly, hemocyanin can be converted into PO-like enzymes in arthropods and mollusks by physical disruption of the structural motifs in the dicopper centers (222), whereas glycosylation modification of hemocyanin or its degradation into functional peptides enhances its antimicrobial activity (223). Despite these findings, it is currently unknown whether the degradation or post-translational modification mechanisms of hemocyanin are regulated by epigenetic inheritance. Thus, further studies would provide better insight into epigenetic reprogramming of the invertebrates’ immune system since such information could be leveraged for designing therapeutic agents for aquaculture invertebrates, such as shrimps, oysters, scallops, etc.

## 7 Conclusion

Arthropods and mollusks have evolved to inherit regulatory mechanisms capable of producing thousands of immune proteins from a few genes by epigenetic modifications, allowing them to recognize and eliminate a wide range of pathogens. These organisms select genes through epigenetics to enhance the recognition of pathogens by expressing specific protein receptors and also modify immune molecules, such as hemocyanin, DSCAM, etc., to perform immune functions, whereas mitochondrial metabolism provides the energy and substrates required for epigenetic modifications. The epigenetics of trained immune memories can last long and be passed on to the next generation. Also, arthropods and mollusks activate the trained immunity response to external stimuli, an immune characteristic that can last for a very long time or even into the next generation. Nonetheless, there is still limited knowledge about pathogen-host interactions, an important factor limiting in-depth trained immunity applications in economically important arthropods and mollusks. Therefore, further research on the mechanism of trained immunity would provide vital information for breeding important economic species, optimize the breeding methods, and speed up the breeding process in arthropods and mollusks.

## Author contributions

JJA and YLZ conceived the idea. MMZ and JJA performed the literature search, wrote the draft, and revised the paper. YLZ and JJA obtained funding and provided supervision. ZL, ZZ, DY, SY, YZ, and XC provided literature input and suggestions. All authors contributed to the article and approved the submitted version.
